# Supplementary material for: Survey and molecular detection of Sri Lankan cassava mosaic virus in Thailand
Source: PLoS One. 2021 Oct 11;16(10):e0252846. doi: 10.1371/journal.pone.0252846 (PMC8504725; doi:10.1371/journal.pone.0252846)
Supplement: S1 Fig — (PDF) [file pone.0252846.s001.pdf]

Fig. S1. Schematic showing the whole-genome sequencing of Sri Lankan cassava mosaic virus (SLCMV) using the primer walking approach.

DNA-A

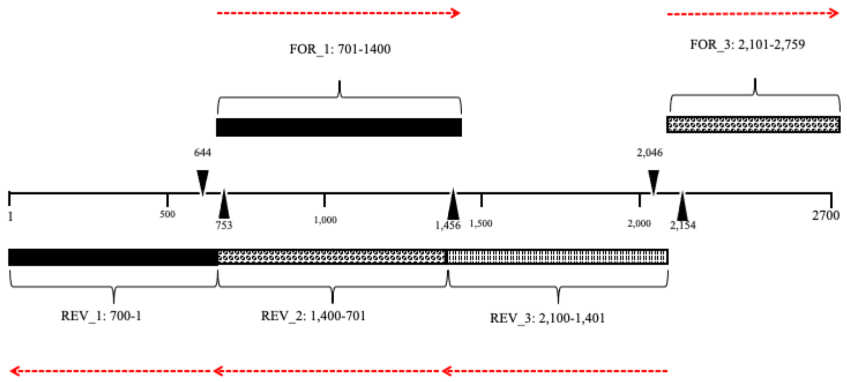

DNA-B

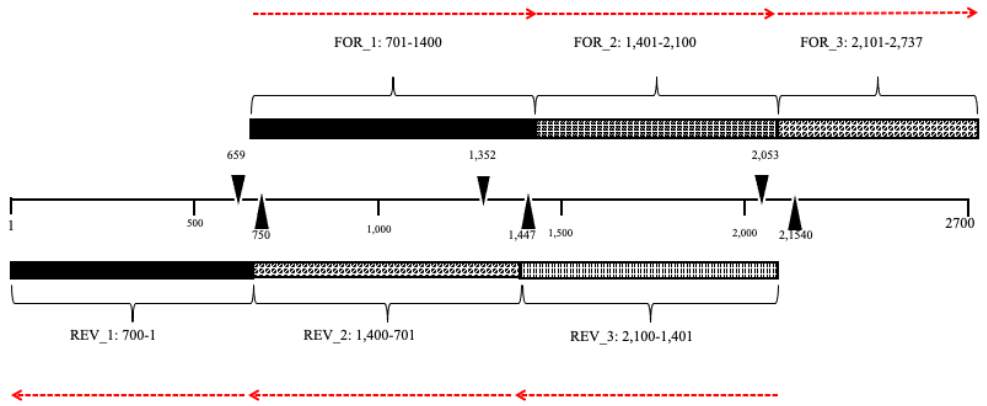

FOR_1: 701-1400
FOR_2: 1,401-2,100
FOR_3: 2,101-2,737
659
1,352
2,053
1
500
750
1,000
1,447
1,500
2,000
2,1540
2700
REV_1: 700-1
REV_2: 1,400-701
REV_3: 2,100-1,401

Table S1. Cassava mosaic disease (CMD) surveillance data collected from Prachinburi, Sakaeo, Burium, Surin, and Sisaket provinces of Thailand, including field location, cassava cultivar, mode of infection, disease severity, and PCR-based detection.

**Primer name**	**Positions**	**Started Site**	**5’→3’**
**DNA-A**			
FOR_1	701-1400	644	CCT GGG TAA GAT ATG GAT GGA
FOR_3	2,101-2,759	2,046	AGG CTG AGA AGG GAG ACA CA
REV_1	700-1	753	CAA AAT CCT GGG GCT TAT CA
REV_2	1,400-701	1,456	ACA GGG GAA CTC ATC ACT GC
REV_3	2,100-1401	2,154	AAG GAG TTA GCA CCC AAG GA
**DNA-B**			
FOR_1	701-1,400	659	GGT ACA GTG TGT ATT AAA AAC AGC CA
FOR_2	1,401-2,100	1,352	AGC CCA ACC TCG CTT CTA AC
FOR_3	2,101-2,737	2,053	CAT AAA GCA AAG CCC AGA GC
REV_1	700-1	750	TCC CGG ACC AAT ACA CAA GT
REV_2	1,400-701	1,447	CCA AGG CCC AAG TCT AAT AGG
REV_3	2,100-1,401	2,140	ATG CCA CGT GGA TGC TCT
