# Supplementary figures and images for: Survey and molecular detection of Sri Lankan cassava mosaic virus in Thailand
Source: PLoS One. 2021 Oct 11;16(10):e0252846. doi: 10.1371/journal.pone.0252846 (PMC8504725; doi:10.1371/journal.pone.0252846)

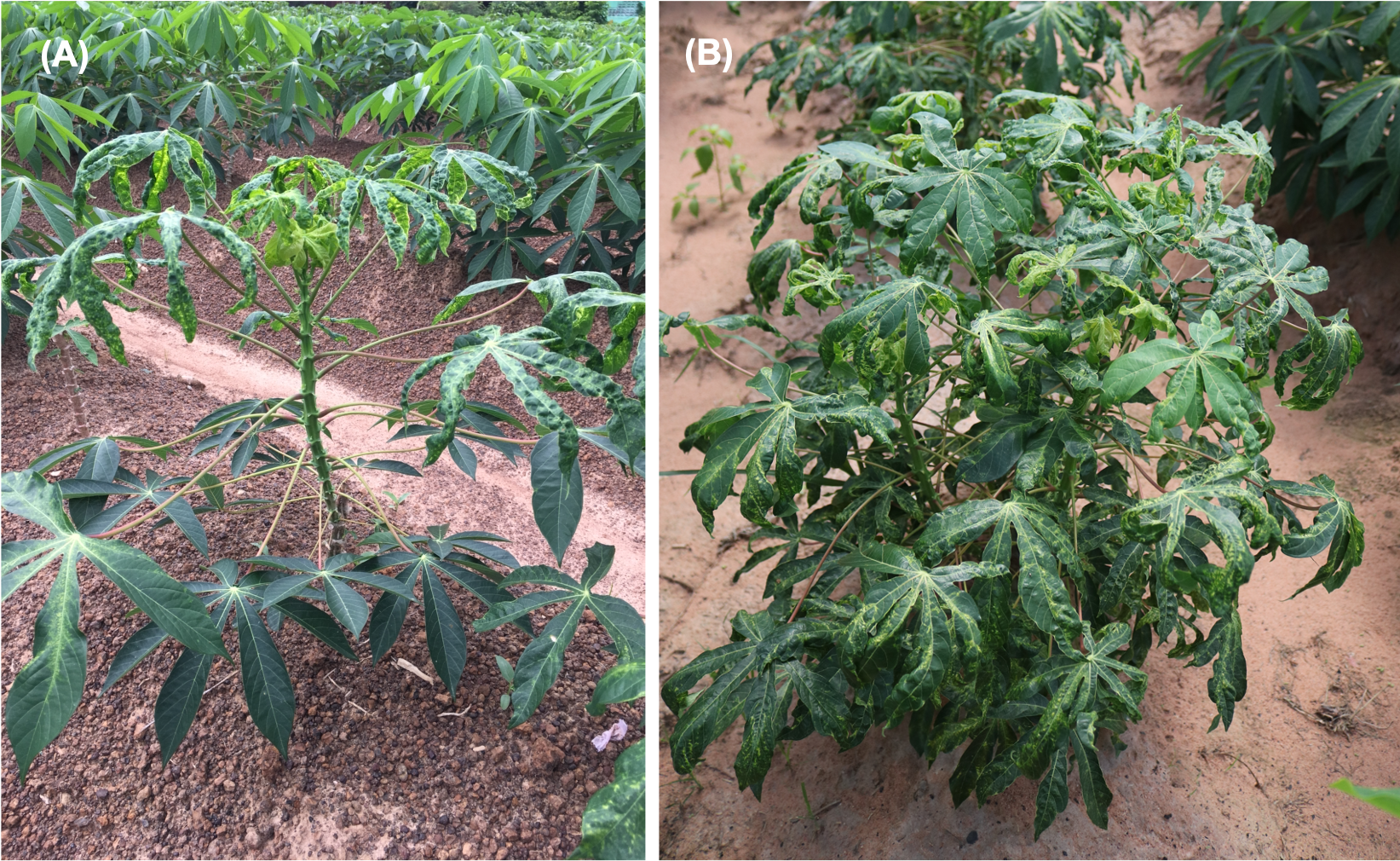

Supplement: S2 Fig — A) CMD transmitted through infected stem, B) CMD transmitted by B. tabaci. (TIF) [file pone.0252846.s002.tif]

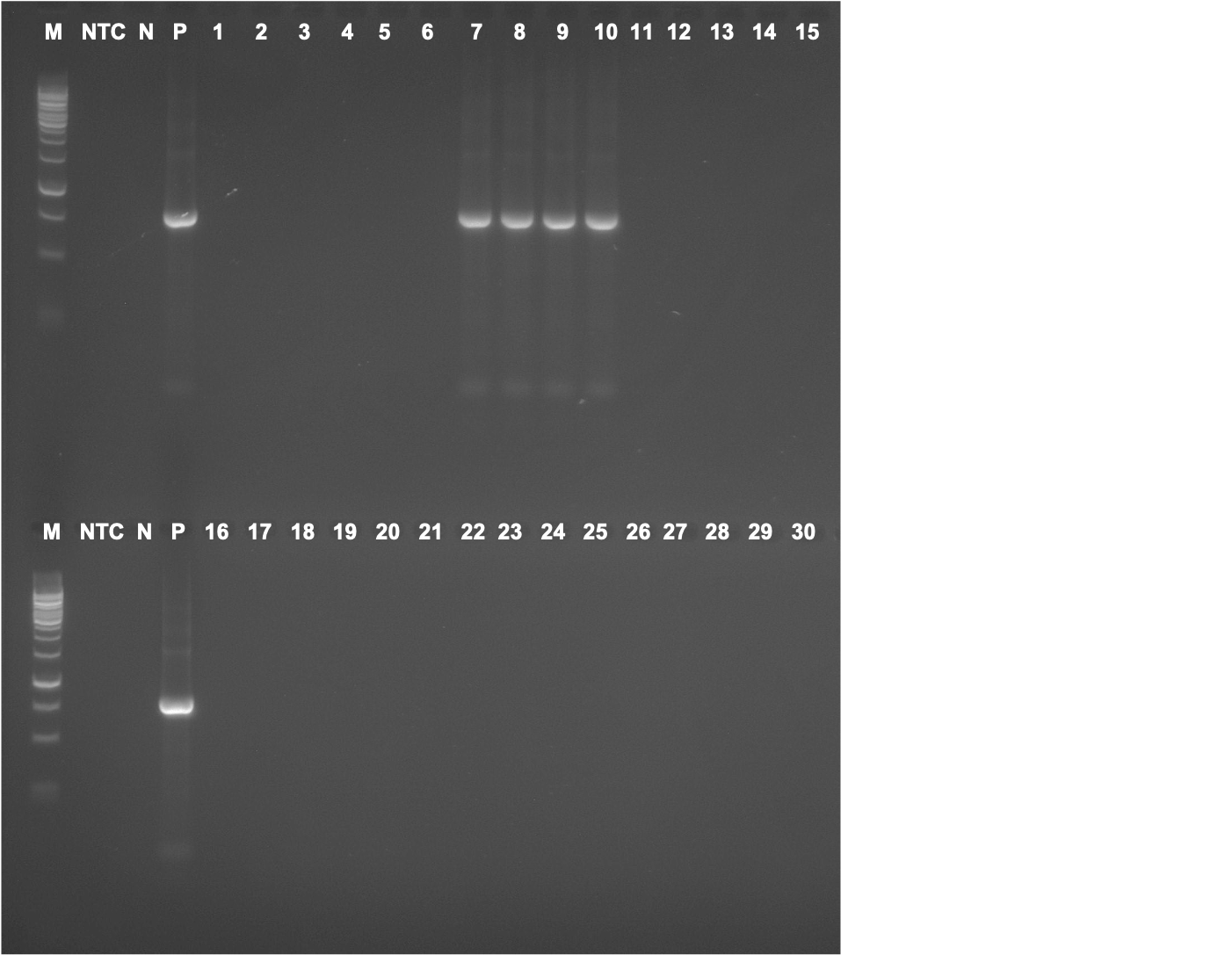

Supplement: S3 Fig — DNA gel electrophoresis of PCR amplification from cassava samples. (TIF) [file pone.0252846.s003.tif]
